# Task-irrelevant spatial dividers facilitate counting and numerosity estimation

**DOI:** 10.1038/s41598-018-33877-y

**Published:** 2018-10-23

**Authors:** Qi Li, Ryoichi Nakashima, Kazuhiko Yokosawa

**Affiliations:** Department of Psychology, Graduate School of Humanities and Sociology, The University of Tokyo, Tokyo, Japan

## Abstract

Counting is characterized as a slow and error-prone action relying heavily on serial allocation of focused attention. However, quick and accurate counting is required for many real-world tasks (e.g., counting heads to ensure everyone is evacuated to a safe place in an emergency). Previous research suggests that task-irrelevant spatial dividers, which segment visual displays into small areas, facilitate focused attention and improve serial search. The present study investigated whether counting, which is also closely related to focused attention, can be facilitated by spatial dividers. Furthermore, the effect of spatial dividers on numerosity estimation, putatively dependent upon distributed attention, was also examined to provide insights into different types of number systems and different modes of visual attention. The results showed profound performance improvement by task-irrelevant spatial dividers in both counting and numerosity estimation tasks, indicating that spatial dividers may activate interaction between number and visual attention systems. Our findings provide the first evidence that task-irrelevant spatial dividers can be used to facilitate various types of numerical cognition.

## Introduction

Enumeration is a basic mathematical ability that supports our actions in a wide range of daily tasks and is closely related to various cognitive functions such as attention^1–5^ and working memory^6,7^. Previous research has provided solid evidence for understanding behavioral characterizations of counting processes. For example, it has been demonstrated that enumeration of a small group, involving four or fewer items, can be achieved in an error-free manner with only a small increase in enumerating time for every additional item (around 50–80 ms/item), while enumeration for larger numerosities (five or more) is more error-prone, with a sharp and linear increase in enumerating time for each extra item (around 200–300 ms/item)^1–3,8–12^. Furthermore, the slope of 200 to 300 ms per item corresponds roughly to the time it takes an adult to recite numbers when counting aloud as fast as possible^13^. The above two types of enumeration are often termed as “subitizing” and “counting”, respectively. In addition to counting, numerosity estimation has been labeled as another type of enumeration of large numerosities. If there is sufficient time, observers may count each item serially to know the exact numerosity; if not, they may estimate quickly but in an approximate fashion, without identify the exact numerosity^14^.

There has been a long-stand debate on mechanisms underlying enumeration. Some researchers proposed that our abilities to subitize small numbers and estimate large numbers are rooted in the same mechanism called the approximate number system (ANS)^15–18^. Evidence supporting this single estimation system hypothesis comes from studies suggesting that the accuracy of numerosity estimation decreases with increasing numerical magnitude according to the Weber fraction^19–23^. For very small numerosities, Weber’s law predicts an excellent discrimination of 1 from 2 from 3, consequently leading to an obvious subitizing effect.

Other researchers argued against the single estimation system hypothesis, and suggested the existence of a dedicated mechanism for apprehending small numerosites, which is sometimes called the precise number system (PNS)^1,3,24–26^. Strong evidence for the PNS hypothesis comes from more recent research that compared estimation precision between two numerosity sets whose discrimination difficulty were matched, and demonstrated a clear violation of Weber’s law, with a much higher precision over numerosities 1–4 comparing to 10–40^27,28^. It is hypothesized that the PNS represents quantities with very high precision, but is severely limited in capacity (<3 or 4 items). These characteristics of PNS can account for the phenomenon of subitizing. Furthermore, in order to identify the exact number of quantities above the subitizing range, it is necessary to count explicitly, which requires effortful shifting of the capacity-limited PNS from one group to the next^29^.

Still other researchers argued that the existence of a third system other than ANS and PNS can contribute to number knowledge. This system is often called the parallel individuation system (PIS) or the object tracking system (OTS)^18,30–33^. PIS represents objects in the visual scene as single unities (“object files”) in working memory, and works by tracking spatiotemporal information, object features, and property change to identify each new object introduced into a scene. There is considerable empirical evidence suggesting that PIS exists in both human (including young children and infant)^34–38^ and nonhuman animals^39,40^. Like PNS, PIS also has severe limits in capacity: it can simultaneously track only about 4 objects. However, it has been shown that observers can exceed their limit of 3 or 4 objects in parallel individuation when a chunking mechanism is available (e.g., 2 clusters of 4 dots can be better enumerated compared to 8 randomly distributed dots)^18^.

Previous studies on enumeration have identified several important mechanisms for understanding human numerical cognition. In recent years, there has been increasing evidence that highlights a role of visual attention in enumeration performance^5,41^, suggesting that there are still other mechanisms that may contribute to numerical cognition. In the present study, we targeted visual attention systems, and aimed to identify situations in which counting and numerosity estimation can be systematically influenced by visual attention. Investigating this issue is theoretically important as it can shed new light on how human number systems function and how they interacts with other cognitive systems. Moreover, revealing conditions that influence counting and numerosity estimation is also of great practical importance. For example, this knowledge may provide new hints to facilitate mathematical development, simplify procedures of tracking quantities of products, and aid relief actions at emergency scenes such as head counting after a fire, explosion, or earthquake.

Recently, Nakashima and Yokosawa^42^ have demonstrated that task-irrelevant spatial dividers which segment the entire display area into smaller areas, significantly improved performance in a serial search task where focused attention is required to scan search items one by one. The authors suggested that spatial dividers may facilitate serial search by inducing a sorting effect on the visual stimuli to form groups, which enables multiple objects (grouped objects) to be processed at one time. This grouping process may consequently increase the efficiency of serial allocation of focused attention. Although serial search and counting seem to be entirely different tasks with distinct task requirements, it is evident that both tasks rely heavily on focused attention to complete some processing steps such as selecting an unprocessed item and marking already processed items. This suggests a possibility that spatial dividers which facilitate deployment and operation of focused attention in a serial task may also have a positive effect on counting processes. The first aim of the current study was to examine this possibility.

Another pertinent finding reported in the same visual search study of Nakashima and Yokosawa concerns the discovery that task-irrelevant spatial dividers interfere with parallel search. Visual search has been a key paradigm in attention research. It is generally agreed that focused attention is required to successively scan items in a search field until the target is identified in a serial search task whereas distributed attention contributes to immediate target detection in a parallel search task^43^. The findings of Nakashima and Yokosawa thus suggest that spatial dividers may differentially impact focused and distributed attention: on the one hand, spatial dividers can facilitate object grouping and aid the process of allocation of focused attention in an inefficient serial search task; on the other hand, redundancy induced by spatial dividers seems to disturb the deployment of distributed attention in an efficient parallel search task. The second aim of the current study was to further investigate the potential effect of spatial dividers on a number perception task involving distributed attention to gain a deeper understanding on how irrelevant visual information modulates number perception and visual attention. To this end, we adopted a numerosity estimation task, which has been suggested by several recent studies to be closely related to the distributed mode of attention^5,11^. If the deployment of distributed attention is disturbed by redundant task-irrelevant information induced by spatial dividers, as is seen in a parallel search task, performance would also be impaired in a numerosity estimation task.

## Experiment 1: Counting Task

In this experiment, we examined whether counting performance can be modulated by task-irrelevant spatial dividers that segment an entire visual display into small areas. Previous research has reported that spatial clustering facilitates serial search^44^. In addition, there is evidence that visual stimuli arranged with high spatial regularity facilitate counting^18^. In the present study, stimuli were distributed relatively evenly throughout the display while the spatial regularity were kept low to minimize potential confounding effects of clustering and spatial regularity.

## Method

### Participants

Twenty students from the University of Tokyo participated in Experiment 1 (aged 19–25, 18 males). All participants reported normal or corrected-to-normal visual acuity, and provided informed consent prior to the experiments. The experiment protocol was approved by the Institutional Review Board of the University of Tokyo. The methods were carried out in accordance with the relevant guidelines and regulations.

### Apparatus

The experiment was conducted in a darkened testing room. Participants sat 75 cm from a 20-inch CRT monitor (60 Hz refresh rate; 1024 × 768 resolution). Visual stimuli were generated using the Psychophysics Toolbox^45,46^ implemented in Matlab.

### Stimuli and design

Stimulus arrays consisting of various numbers of white circles (diameter = 0.6°) were presented within a 12.6° × 12.6° region with a gray background (9.5 cd/m^2^). There were five target numerosities (13, 20, 27, 34 and 41 circles) and sixteen filler numerosities (12, 14, 17, 19, 21, 23, 26, 28, 30, 33, 35, 38, 40, 42, 45 and 47 circles). Filler trials were included to ensure the unpredictability of numerosities.

**Figure 1:**
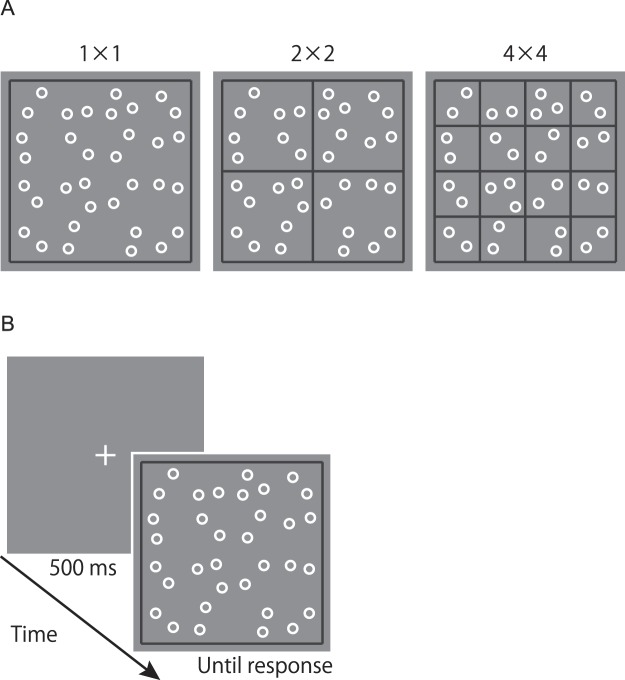
(**A**) Example displays for the three divider conditions. (**B**) The sequence of trial events in Experiment 1.

Spatial dividers in this study were black lines with a width of 0.2°. Figure 1A shows the three divider conditions used in this study; they differ in the number of rows and columns determined by the dividers. In the 1 × 1 divider condition, participants were presented with a single large frame surrounding the entire array (12.6° × 12.6°). This condition served as a baseline measure of performance. In the 2 × 2 divider condition, the counting display contained a frame that segregated the large stimulus region into four smaller regions (each subdivided area subtended 6.2° × 6.2°). In the 4 × 4 divider condition, the entire stimulus region was divided into sixteen smaller regions (each subdivided area subtended 3° × 3°). In all divider conditions, circles were placed within a 4 × 4 grid (coincided with the 4 × 4 divider, but was invisible in the 1 × 1 and 2 × 2 divider condition). The position of each circle was randomly selected from 64 slightly jittered possible positions with the following constraints. For numerosities less than 16, each cell of the 4 × 4 grid contained zero or one circle. For numerosities between 16 and 32, each cell contained one or two circles. For numerosities larger than 32, each cell contained two or three circles. As mentioned before, visual displays without clustering and spatial regularity were preferred in the current study to avoid potential confounding effects. The constraints used here served to distribute stimuli relatively evenly throughout the display while keeping the spatial regularity low.

### Procedure

Participants started each trial by pressing a key on the numeric keypad. As shown in Figure 1B, trials began with a white central fixation cross (“+”, 0.6° × 0.6°) lasting 500 ms. The fixation display was followed by a counting display consisting of an array of white circles. The counting display remained present until the response key (the “5” key on the numeric keypad) was pressed, and it was then replaced by a response display indicating participants to report the number of circles orally. Participants were asked to press the response key as soon as they finished counting. They were also instructed to count the number of the circles as quickly and accurately as possible.

The experiment consisted of a total of 288 trials in total (96 trials for each of the three divider conditions). In each divider condition, there were 16 trials for any of the five target numerosities, and one trial for any of the sixteen filler numerosities. All conditions occurred in a random order throughout the experiment. Participants completed 15 practice trials (5 trials for each of the three divider conditions) before the actual experiment. Numerosities used in the practice were 15, 22, 29, 36, and 43, which were different to those used in the main experiment. During the practice session, the accurate numerosity was displayed as a feedback after each trial. No feedback was given during the experimental session.

### Data analysis

Response times (RTs) that fell outside three standard deviations from the individual mean for every combination of condition were removed as outliers. Only correct trials were used in the RT analysis. RTs on target trials were analyzed using a two-way repeated measures analyses of variance (ANOVA) with divider (1 × 1, 2 × 2, 4 × 4) and numerosity (13, 20, 27, 34, 41) as within-subjects factors. Accuracy on target trials was also analyzed using a similar 3 × 5 ANOVA as that used for RT data. Shaffer’s modified sequential rejective Bonferroni procedure was used for multiple comparisons.

## Results and Discussion

### RTs

**Figure 2:**
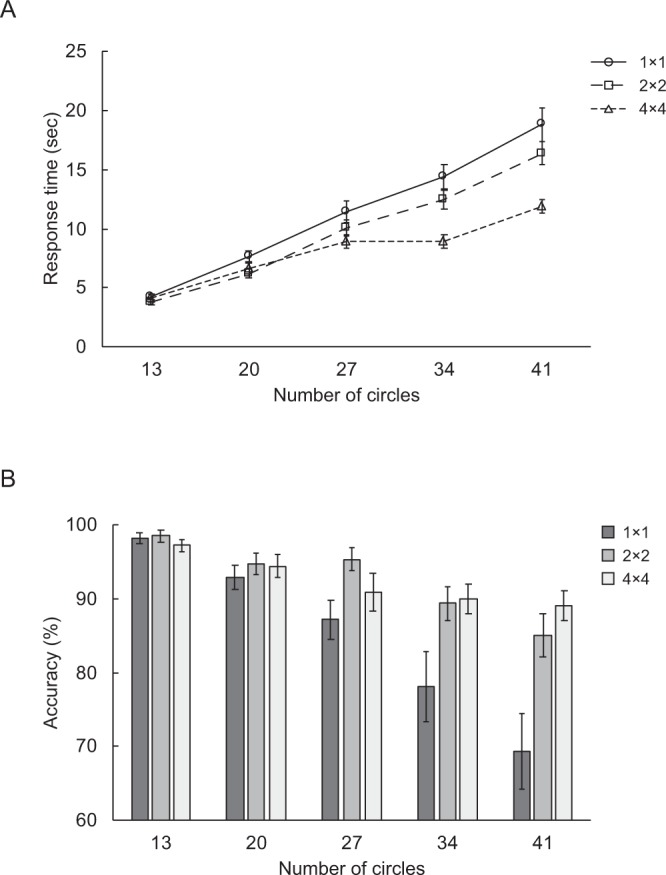
(**A**) Mean RT and (**B**) mean accuracy for target numerosities, separated according to the factor of divider. Error bars represents standard errors.

Figure 2A shows mean RTs for target numerosities. RT advantages for the 2 × 2 and 4 × 4 divider conditions relative to the baseline 1 × 1 divider condition were tested by a two-way repeated-measures ANOVA with factors of divider (1 × 1, 2 × 2, 4 × 4) and numerosity (13, 20, 27, 34, 41). There was a significant main effect of divider (*F*(2,38) = 23.93, *p* < 0.001, *η*_*p*_^2^ = 0.56), a significant main effect of numerosity (*F*(4,76) = 199.17, *p* < 0.001, *η*_*p*_^2^ = 0.91), and a significant interaction between divider and numerosity (*F*(8,152) = 15.44, *p* < 0.001, *η*_*p*_^2^ = 0.45). Follow-up tests of simple effects for the interaction showed that the effect of divider was significant in all numerosity conditions (numerosity 13: *F*(2,38) = 3.86, *p* = 0.030, *η*_*p*_^2^ = 0.17; numerosity 20: *F*(2,38) = 12.37, *p* < 0.001, *η*_*p*_^2^ = 0.39; numerosity 27: *F*(2,38) = 5.67, *p* = 0.007, *η*_*p*_^2^ = 0.23; numerosity 34: *F*(2,38) = 19.78, *p* < 0.001, *η*_*p*_^2^ = 0.51; numerosity 41: *F*(2,38) = 29.33, *p* < 0.001, *η*_*p*_^2^ = 0.61), reflecting that spatial dividers affected counting time. Multiple comparisons revealed that the effect of spatial dividers varies across numerosities. In the numerosity 13 condition, 1 × 1 trials had significantly longer RTs than 2 × 2 trials (*t*(19) = 3.85, *p* = 0.003). There was no significant difference between 2 × 2 and 4 × 4 trials (*t*(19) = 1.73, *p* = 0.100), or between 1 × 1 and 4 × 4 trials (*t*(19) = 0.73, *p* = 0.477). In the numerosity 20 and 27 conditions, 1 × 1 trials had significantly longer RTs than both 2 × 2 and 4 × 4 trials (numerosity 20: 1 × 1 vs. 2 × 2: *t*(19) = 4.67, *p* < 0.001; 1 × 1 vs. 4 × 4: *t*(19) = 2.98, *p* = 0.008; numerosity 27: 1 × 1 vs. 2 × 2: *t*(19) = 2.23, *p* = 0.043; 1 × 1 vs. 4 × 4: *t*(19) = 2.70, *p* = 0.043). The difference between 2 × 2 and 4 × 4 trials was marginally significant for numerosity 20 (*t*(19) = 1.90, *p* = 0.072), but did not approach significance for numerosity 27 (*t*(19) = 1.72, *p* = 0.101). In the numerosity 34 and 41 conditions, RTs were longest on 1 × 1 trials, followed by 2 × 2 trials, and were fastest on 4 × 4 trials (numerosity 34: 1 × 1 vs. 4 × 4: *t*(19) = 5.09, *p* < 0.001; 1 × 1 vs. 2 × 2: *t*(19) = 2.67, *p* = 0.015; 2 × 2 vs. 4 × 4: *t*(19) = 4.38, *p* < 0.001; numerosity 41: 1 × 1 vs. 4 × 4: *t*(19) = 5.97, *p* < 0.001; 1 × 1 vs. 2 × 2: *t*(19) = 3.25, *p* = 0.004; 2 × 2 vs. 4 × 4: *t*(19) = 5.72, *p* < 0.001). These results revealed that spatial dividers reduce counting time. Moreover, the effect of spatial dividers seems more pronounced in large numerosity conditions, plausibly because RTs in the small numerosity conditions was close to ceiling.

In addition, RTs significantly slowed as numerosity increased in all divider conditions (1 × 1: *F*(4,76) = 85.00, *p* < 0.001, *η*_*p*_^2^ = 0.82; 2 × 2: *F*(4,76) = 174.79, *p* < 0.001, *η*_*p*_^2^ = 0.90; 4 × 4: *F*(4,76) = 97.03, *p* < 0.001, *η*_*p*_^2^ = 0.84). There were significant differences between all numerosity conditions (all *p*s < 0.001), except between numerosities 27 and 34 in the 4 × 4 divider condition (*p* = 0.909). The results that RTs increased with the number of counting items is consistent with previous research suggesting that counting involves serial processing^3,5^.

### Accuracy

Figure 2B shows mean accuracy for target numerosities. Accuracy advantages for the 2 × 2 and 4 × 4 divider conditions relative to the baseline 1 × 1 divider condition were tested by a two-way repeated-measures ANOVA with factors of divider (1 × 1, 2 × 2, 4 × 4) and numerosity (13, 20, 27, 34, 41). There was a main effect of divider (*F*(2,38) = 10.39, *p* < 0.001, *η*_*p*_^2^ = 0.35), a main effect of numerosity (*F*(4,76) = 28.46, *p* < 0.001, *η*_*p*_^2^ = 0.60), and an interaction between divider and numerosity (*F*(8,152) = 4.65, *p* < 0.001, *η*_*p*_^2^ = 0.20). Follow-up tests of simple effects revealed that this interaction was due to the fact that the effect of divider was significant in large numerosity conditions (numerosity 27: *F*(2,38) = 4.89, *p* = 0.013, *η*_*p*_^2^ = 0.20; numerosity 34: *F*(2,38) = 4.46, *p* = 0.018, *η*_*p*_^2^ = 0.19; numerosity 41: *F*(2,38) = 10.61, *p* < 0.001, *η*_*p*_^2^ = 0.36), but was not significant in small numerosity conditions (numerosity 13: *F*(2,38) = 0.64, *p* = 0.534; numerosity 20: *F*(2,38) = 0.54, *p* = 0.587). As can be seen in Figure 2B, even in the baseline 1 × 1 divider condition, accuracy scores were very high on small numerosity trials (numerosity 13: Mean = 98.12, SE = 0.78; numerosity 20: Mean = 92.81, SE = 1.67). It is likely that the lack of an effect of divider is due to a ceiling effect in the small numerosity conditions. Multiple comparisons for the effect of divider on large numerosities further revealed that, accuracy was significantly worse on 1 × 1 divider trials compared to 2 × 2 divider trials in the numerosity 27 condition (*t*(19) = 2.98, *p* = 0.023). In the numerosity 34 condition, 1 × 1 divider trials had slightly worse accuracy than both 2 × 2 and 4 × 4 divider trials (1 × 1 vs. 2 × 2: *t*(19) = 2.34, *p* = 0.091; 1 × 1 vs. 4 × 4: *t*(19) = 2.32, *p* = 0.091). In the numerosity 41 condition, 1 × 1 divider trials had significantly worse accuracy than both 2 × 2 and 4 × 4 divider trials (1 × 1 vs. 2 × 2: *t*(19) = 3.02, *p* = 0.007; 1 × 1 vs. 4 × 4: *t*(19) = 4.17, *p* = 0.002). These findings indicate that the precision of counting is improved by spatial dividers.

Because both RT and accuracy data showed behavioral advantages of spatial dividers, we can rule out explanations based on speed-accuracy tradeoffs. The results thus suggest that counting time and counting precision can be substantially influenced by task-irrelevant spatial dividers. Our findings are consistent with previous research showing that spatial dividers aid serial search^42^, and provide further evidence supporting the view that the focal mode of visual attention can be effectively modulated by task-irrelevant spatial information.

## Experiment 2: Numerosity Estimation Task

The relationship between distributed attention and the ability to estimate an approximate number has been highlighted by several lines of recent research^5,11^. In Experiment 2, we used a numerosity estimation task to examine whether the behavioral benefits of spatial dividers observed in Experiment 1 are confined to serial counting. Because previous research has reported that task-irrelevant spatial dividers interfere with parallel search^42^, suggesting that distributed attention is disturbed by visual redundancy. We thus predicted that numerosity estimation performance would be impaired by task-irrelevant spatial dividers.

## Method

Unless stated otherwise, the method in Experiment 2 was identical to that of Experiment 1.

### Participants

A group of 20 new participants (aged 19–25, 15 males) took part in the experiment.

### Procedure

**Figure 3:**
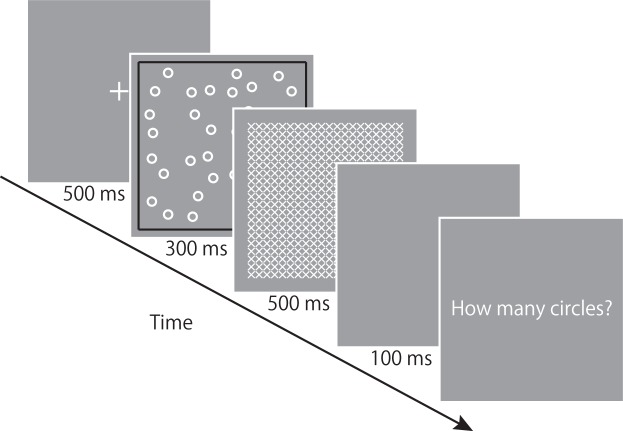
The sequence of trial events in Experiment 2.

The trial sequence is shown in Figure 3. Each trial included the following events: a central fixation for 500 ms, an array of white circles for 300 ms, a mask display consisting of uniformly distributed white X-shaped crosses covering an area of 12.6° × 12.6° for 500 ms, a blank gray screen for 100 ms, and a response display that lasted until a particular key was pressed. Participants were instructed to estimate as accurately as possible the number of circles, and to report the estimated value orally. After the oral response, participants pressed the “5” key on the numeric keypad to proceed to the next trial.

### Data analysis

The precision of participants’ numerosity estimations was measured by a difference score, which was calculated by subtracting the exact value from the estimated value. Positive scores indicate overestimation, whereas negative scores indicate underestimation. Difference scores on target trials were analyzed using a two-way ANOVA with divider (1 × 1, 2 × 2, 4 × 4) and numerosity (13, 20, 27, 34, 41) as within-subjects factors.

## Results and Discussion

**Figure 4:**
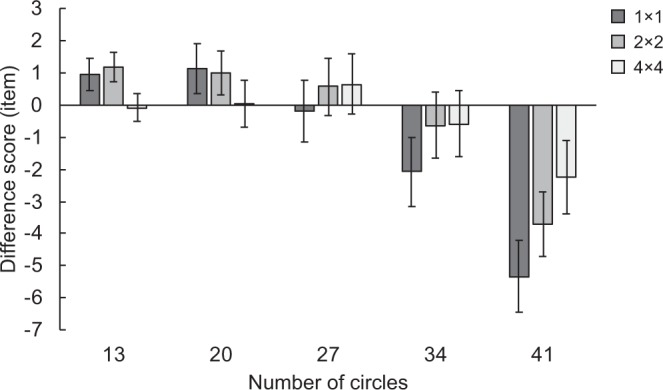
Mean difference scores for target numerosities, separated according to the factor of divider. Error bars represent standard errors.

As illustrated in Figure 4, mean difference scores (estimated value - exact value) approach to a floor of zero in all divider conditions for numerosities 13, 20 and 27, but seem to differ largely between baseline and the two remained divider conditions for numerosities 34 and 41. A 3 (divider) ×5 (numerosity) ANOVA was conducted on difference scores. There was a marginally significant main effect of divider (*F*(2,38) = 2.88, *p* = 0.069, *η*_*p*_^2^ = 0.13), a significant main effect of numerosity (*F*(4,76) = 23.07, *p* < 0.001, *η*_*p*_^2^ = 0.55), and a significant interactions between these two factors (*F*(8,152) = 10.14, *p* < 0.001, *η*_*p*_^2^ = 0.35). Follow-up tests of simple main effects for the interaction revealed that the effect of divider was significant for numerosities 13, 20, 34, and 41 (numerosity 13: *F*(2,38) = 8.83, *p* < 0.001, *η*_*p*_^2^ = 0.32; numerosity 20: *F*(2,38) = 3.85, *p* = 0.030, *η*_*p*_^2^ = 0.17; numerosity 34: *F*(2,38) = 3.37, *p* = 0.045, *η*_*p*_^2^ = 0.15; numerosity 41: *F*(2,38) = 14.43, *p* < 0.001, *η*_*p*_^2^ = 0.43), but did not approach significance for numerosity 27 (*F*(2,38) = 2.07, *p* = 0.140). The significant simple main effects of divider were further analyzed by pairwise comparisons and revealed different patterns in different numerosity conditions. In the numerosity 13 condition, difference scores for 4 × 4 divider trials differed significantly from both 1 × 1 and 2 × 2 divider trials (1 × 1 vs. 4 × 4: *t*(19) = 3.73, *p* = 0.004; 2 × 2 vs. 4 × 4: *t*(19) = 3.34, *p* = 0.004). Similarly, in the numerosity 20, there was a significant difference between 4 × 4 and 1 × 1 divider trials (*t*(19) = 3.46, *p* = 0.008), and a difference that approached significance between 4 × 4 and 2 × 2 divider trials (*t*(19) = 1.85, *p* = 0.080). The results in the numerosity 13 and 20 conditions suggest that the extent of overestimation was less on 4 × 4 divider trials than 1 × 1 and 2 × 2 divider trials in small numerosity conditions. In the numerosity 34 condition, there was a significant difference between 1 × 1 and 2 × 2 divider trials (*t*(19) = 2.75, *p* = 0.039), and a difference that approached significance between 1 × 1 and 4 × 4 divider trials (*t*(19) = 2.03, *p* = 0.057). In the numerosity 41 condition, difference scores were lowest on 1 × 1 divider trials, followed by 2 × 2 divider trials, and were highest on 4 × 4 divider trials (1 × 1 vs. 4 × 4: *t*(19) = 4.17, *p* = 0.002; 1 × 1 vs. 2 × 2: *t*(19) = 3.50, *p* = 0.002; 2 × 2 vs. 4 × 4: *t*(19) = 3.07, *p* = 0.006). The results in the numerosity 34 and 41 conditions revealed that the number of stimuli was underestimated less when task-irrelevant spatial dividers were presented. Taken together, the degree of overestimation in small numerosity conditions was reduced by the 4 × 4 spatial divider, and the degree of underestimation in large numerosity conditions was reduced by both 2 × 2 and 4 × 4 spatial dividers. Note that difference scores for numerosities 13, 20, and 27 were very low even in the baseline 1 × 1 divider condition (numerosity 13: *M* = 0.93 item; numerosity 20: *M* = 1.13 item; numerosity 27: *M* = −0.20 item). This indicates that our participants were able to estimate with high precision up to 27 items, which means there was little room for improvement in small numerosity conditions. This may cause the relatively limited effects of spatial dividers on estimation precision in numerosities13 and 20 conditions, as well as a lack of effect of spatial dividers in the numerosity 27 condition. However, in large numerosity conditions where estimation was not precise in the baseline 1 × 1 divider condition (numerosity 34: *M* = −2.09 item; numerosity 41: *M* = −5.33 item), not only the 4 × 4 spatial dividers, but also the 2 × 2 spatial dividers exerted effects on estimation precision.

One may ask why our participants were capable to estimate highly accurately in numerosity 13, 20 and 27 conditions where the number of objects goes beyond the limits of our parallel enumeration abilities (no more than four items). It has long been known that one single exposure to veridical numerical information, are sufficient to calibrate the estimation system. For example, presenting observers with a set of two hundred dots labeled as such improved their estimation of sets between ten and four hundred dots^13^. In the current experiment, our participants received feedback of the exact number of circles during the practice phase. It is highly plausible that our participants took advantage of the feedback to calibrate their estimation system.

The present findings of facilitative effects of task-irrelevant spatial dividers were opposite to our original predictions; they also contradict previous research using a parallel search task. Consequently, our findings suggest that the effect of spatial dividers on distributed attention is crucially dependent on task requirements. In a numerosity estimation task, participants can exploit redundant visual information to achieve more accurate estimation.

## General Discussion

The ability to perceive and manipulate numerical information is crucial for a wide variety of daily activities. When perceiving large numerosities, we can either count the elements to determine the exact value, or make a rough estimation in a glance. In the present study, we investigated whether and how spatial dividers influence counting and numerosity estimation. Our results provide the first evidence that task-irrelevant spatial dividers facilitate both counting and numerosity estimation. In the following section, we detail possible mechanisms underlying the facilitative effects on counting and estimating performance in the context of human number systems and visual attention.

### Spatial dividers activate subitizing and/or chunking

Some theories on numerosity perception and numerical cognition assume two different types of number systems: PNS and ANS^3^. PNS functions in a rapid and error-free manner, but it is subject to the limitation that it can handle no more than four objects. On the other hand, ANS can operate over larger numerosities in a fast but inaccurate manner. These two systems allow subitizing of small numerosities and estimation of large numerosities, respectively. To reach high precision beyond the subitizing range, counting is required, which involves item-by-item processing and relies heavily on serial shifts of focused attention. Still, another hypothesis claimed that an efficient mechanism based on parallel individuation (i.e., PIS) is responsible for the phenomenon of subitizing^18,30–33^. However, similar to PNS, parallel individuation also has a processing bottleneck of about 3 or 4 objects. Although it has been proposed that this bottleneck can be partially bypassed by involving a chunking mechanism, empirical research has suggested that chunks comprising five or more items would be too difficult to appreciate reliably in a precise manner^18^. It is important to note that observers may be capable of generating a chunk having five or more objects, but it seems difficult to precisely identify the exact number of items in each chunk in this case.

The numerosities used in our experiments went far beyond the processing capacity limit of PNS as well as PIS, and thus these efficient but capacity-limited systems seem unlikely to operate in the baseline condition. However, they could have plausibly been activated when the visual displays were subdivided into smaller regions that contained a smaller number of items (four items or less). This means that, in conditions where the numbers of objects in each subdivided area were within the subitizing range, observers were capable to apprehend the number of items at a glance. Alternatively, spatial dividers may facilitate chunking by grouping objects belonging to the same subdivided areas as a single unit, and the exact number of items in each chunk is available when there are no more than four items in each subdivided area. In the counting task, participants could take advantage of subitizing and chunking to reduce both perceptual load (e.g., individuation of each item) and working memory load (e.g., summing and maintaining the cumulative total of items). In the numerosity estimation task, PNS and/or PIS activated by spatial divider may have helped complete ANS by providing accurate local quantitative information.

Although explanations based on subitizing and chunking account for a large part of the facilitative effects of spatial dividers in conditions where subdivided areas comprising no more than four items, it is unlikely that they are the only mechanisms that were mediated by spatial dividers. In fact, there was clear evidence for behavioral benefits of dividers even when the number of items in each subdivided area exceeded the subitizing range. For example, benefits in both counting and estimating were observed on numerosity 34 and 41 trials when 2 × 2 spatial dividers were presented. In these conditions, the number of circles in each subdivided area ranged between 8 and 11. The subitizing and chunking accounts do not adequately explain the behavioral advantages in these conditions. Empirical evidence coming from the field of visual search suggests that task-irrelevant spatial dividers segmenting visual displays into small regions can influence the deployment of visual attention^42^. The observed effects of spatial dividers on counting and estimation performance, especially in conditions where subitizing and chunking mechanisms were not available or sufficient, plausibly reflect that visual attention systems are affected in a way that facilitate counting and estimation.

### Spatial dividers facilitate the allocation of focused attention when counting

Counting large numerosites is a complex process that typically involves a series of processing stages such as individuating and localizing each element in the visual field, summing the number of the elements, maintaining a running total of these elements, and marking a just counted element to inhibit recounting. In general, this process is slow, effortful, and error-prone because it requires serial allocation of focused attention. In our counting experiment (Experiment 1), spatial dividers may guide and facilitate the allocation of focused attention by providing organizational structure and processing order. This allows participants to count more efficiently and accurately when compared to situations lacking such guidance. In addition, spatial dividers may serve as marking cues that help participants avoid revisiting an already visited region. Revisit errors to previously inspected locations can therefore be diminished. The possible mechanisms described above can be examined by recording eye movements. Future research using eye tracking is required to provide further insights.

### Spatial dividers mediate distributed attention when estimating

As mentioned previously, the ability to estimate numerosities is generally assumed to rely on ANS that enables an approximate representation of numerical magnitude^15–18^. A recent study showed that the ability to estimate numerical quantities was profoundly improved for patients with simultanagnosia when elements were arranged in ways that facilitated grouping^11^. The authors suggested that grouping may enable all elements to be coded and compared together, resulting in facilitation of magnitude estimation. It is plausible that such a grouping mechanism plays a role in our estimation experiment. That is, spatial dividers may stimulate grouping and thereby enable magnitude comparison between groups when participants adopt the distributed mode of attention. Magnitude comparison provides additional information about quantitative relationships between subdivided areas; in turn, this information can be used to correct an initial estimate. Moreover, participants in the present experiment were allowed to use computational skills when the visual field was divided. For example, they could estimate the number of elements in a subdivided region, multiply by the number of regions, and adjust the outcome based on fast magnitude comparison between subdivided regions. Thus, improved estimation precision by spatial dividers may reflect the difference between estimating one numerous group and estimating several less numerous groups. In fact, underestimation is typically observed in a numerosity estimation tasks, and the extent of underestimation is known to increase with increasing numerosities^47,48^. In our estimation experiment (Experiment 2), underestimation was consistently observed for large numerosities (numerosities 34 and 41), and the extent of underestimation greatly decreased in the 2 × 2 and 4 × 4 divider conditions compared to the baseline condition. These findings support the idea that spatial dividers reduce underestimation by generating representations of less numerous groups.

### Comparison with previous research using visual search tasks

A previous study has reported facilitative and interference effects of spatial dividers on serial and parallel searches, respectively^42^. Our results revealed both similarities and differences in the effects of spatial dividers between numerosity perception and visual search. The benefits of spatial dividers in a counting task support the idea that spatial dividers generally facilitate the focal mode of attention. Although counting and serial search present quite different task demands, successful performance in both tasks requires observers to adopt the focal mode of attention to select visual objects in turn, and mark the previously inspected objects. Thus, increased efficiency in allocating focused attention may lead to improved performance in both tasks. On the other hand, the advantage of presenting spatial dividers in the numerosity estimation task seems to contradict results from previous research which has found a small interference effect of spatial dividers in a parallel search task^42^. In this previous research, the authors suggested that task-irrelevant spatial dividers create redundancy that interferes with early-stage visual processing. The implication from their findings is that the distributed mode of attention which operates in parallel and covers the whole visual display is disturbed by spatial dividers. It is plausible that even in the present study, some functions of distributed attention were inhibited by spatial dividers. However, other functions of distributed attention (e.g., magnitude comparison between different groups), which are critical for numerosity perception, might have been activated by spatial dividers. For example, spatial dividers may actually increase difficulties in estimating the total quantity of the whole display, while also activating magnitude comparisons between subdivided areas. As a result, regardless of a disturbance of the process of estimating the quantity of elements distributed across the whole display, participants can adopt a strategy of estimating the magnitude of a small region, comparing it with the other regions, and using computational skills to complete the estimation task. Because precise estimation is typically more common with small than large numbers^47,48^, the above strategy can increase estimation precision, especially for large numerosities. Taken together, differential effects of spatial dividers on numerosity estimation and parallel search may reflect that different functions of distributed attention were mediated in the two tasks.

## Conclusions

We have provided the first evidence that spatial dividers improve counting and numerosity estimation performance. Our findings suggest that spatial dividers can effectively influence focused and distributed attention. Moreover, our findings demonstrate that counting and numerosity estimation are processes with considerable flexibility in adjusting strategies to different situations.

## Data Availability

The datasets generated or analyzed during the current study are available from the corresponding author on reasonable request.
